# Predictors of visual outcome after pars plana vitrectomy secondary to proliferative diabetic retinopathy

**DOI:** 10.22336/rjo.2023.46

**Published:** 2023

**Authors:** Faruk Nisic, Aida Pidro Gadzo, Almir Fajkic, Aida Nisic, Ajla Pidro Miokovic, Goran Damjanovic, Edin Begic, Nermina Beslic, Orhan Lepara

**Affiliations:** * Ophthalmology Clinic, University Clinical Center Sarajevo, Sarajevo, Bosnia and Herzegovina; ** Ophthalmology Department, “Prim. Dr. Abdulah Nakas” General Hospital, Sarajevo, Bosnia and Herzegovina; *** Department of Pathophysiology, Faculty of Medicine, University of Sarajevo, Bosnia and Herzegovina; **** Specialty Consultative Health Care of PI Health Centre of Sarajevo Canton, Sarajevo, Bosnia and Herzegovina; ***** Ophthalmological Polyclinic Vukas Zagreb, Zagreb, Croatia; ****** Clinic for Eye Disease, Clinical Center Serbia, Belgrade, Serbia; ******* Department of Cardiology, “Prim. Dr. Abdulah Nakas” General Hospital, Sarajevo, Bosnia and Herzegovina; ******** Department of Nuclear Medicine and Endocrinology, University Clinical Center Sarajevo, Bosnia and Herzegovina; ********* Department of Human Physiology, Faculty of Medicine, University of Sarajevo, Bosnia and Herzegovina

**Keywords:** diabetic retinopathy, pars plana vitrectomy, visual outcome

## Abstract

**Objective:** Advanced proliferative diabetic retinopathy can lead to serious ophthalmological complications, including blindness. This research aimed to determine visual outcomes after pars plana vitrectomy secondary to proliferative diabetic retinopathy, as well as to identify its predictors.

**Methods:** This prospective clinical study was performed in the Ophthalmology Clinic of the Clinical Centre University of Sarajevo. 60 subjects (eyes) with performed pars plana vitrectomy secondary to proliferative diabetic retinopathy were included in the study.

**Results:** After univariate linear regression analysis, glucose, HbA1c, vascular endothelial growth factor, previous pan-retinal laser photocoagulation, baseline best corrected visual acuity, gas injection, vitreous haemorrhage, iris rubeosis, and glaucoma were found to be statistically significant parameters associated with postoperative visual outcome (p<0.05). Multivariate linear regression analysis was performed to evaluate the association between factors and postoperative best corrected visual acuity. Only intravitreal vascular endothelial growth factor concentration, previous pan-retinal photocoagulation, and gas injection remained statistically significant associated with postoperative best corrected visual acuity (p<0.05).

**Conclusion:** Vitrectomy is an effective treatment for advanced proliferative diabetic retinopathy. Factors correlated with the better visual outcome are good systemic control, previous pan-retinal photocoagulation, low intravitreal vascular endothelial growth factor concentration, younger age, intraoperative internal gas tamponade, combined phacoemulsification and pars plana vitrectomy surgery, and the absence of postoperative complications.

**Abbreviations**: PDR = proliferative diabetic retinopathy, VEGF = vascular endothelial growth factor, TDR = tractional retinal detachment, BCVA = best corrected visual acuity, DR = diabetic retinopathy, RDD = rhegmatogenous retinal detachment, NVG = neovascular glaucoma, BRVO = branch retinal vein occlusion, CBC = complete blood count, DBT = differential blood count, ESR = erythrocyte sedimentation rate, HbA1c = glycosylated hemoglobin, PHACO = phacoemulsification, ILM = internal limiting membrane, PPV = pars plana vitrectomy, IOP = intraocular pressure, PRP = pan-retinal photocoagulation, ETDRS = Early treatment diabetic retinopathy study

## Introduction

Proliferative diabetic retinopathy (PDR) is a frequent indication for PPV surgery. The incidence of blindness in PDR patients has decreased in the last decades due to improved health care of diabetic patients, more approachable advanced ophthalmological care (laser photocoagulation and anti-VEGF treatment), and regular ophthalmological follow-ups [**1**]. Advanced stages of PDR include vitreous haemorrhage and tractional retinal detachment (TRD) and are usually connected to poor outcomes, including lower best-corrected visual acuity (BCVA) and poor anatomical outcome [**1**].

The indications for PPV in diabetic retinopathy (DR) patients are recurrent and persistent vitreous haemorrhage, premacular haemorrhage, TRD involving the macula, combined tractional and rhegmatogenous retinal detachment (TRD/RDD), ghost cell glaucoma, cataract with severe PDR, and iris rubeosis with opaque media [**2**]. The main goal of the surgery is to remove fibrovascular retinal lesions and restore vision or at least to prevent blindness [**3**]. Restoring useful vision is of immense importance since the average age of patients who undergo PPV is 40-60 years [**4**] and their life expectancy is approximately 70 years [**5**]. Therefore, the vision loss of these patients will lead to high social, economic, and healthcare burdens. Delayed visual recovery after PPV can be due to postoperative complications such as vitreous haemorrhage, neovascular glaucoma (NVG), and RD, which sometimes need additional surgery [**6**]. In addition, younger PDR patients have more decreased visual acuity, worse anatomical outcomes after PPV, and a higher postoperative complication rate than older patients [**7**].

Most other studies have reported anatomical outcomes [**8**], but few report visual outcomes after PPV in diabetic retinopathy patients. Thus, this research aims to determine visual outcomes after PPV secondary to PDR and identify its predictors. 

## Methods

This prospective clinical study was performed at the Clinical Centre University of Sarajevo. 60 subjects (eyes) with PPV secondary to PDR were included in the study. All patients underwent the same diagnostics algorithm, which included detailed anamnestic examination, BCVA, intraocular tonometry using Goldman applanation tonometry, slit-lamp anterior and posterior segment examination, and A and B scan ultrasonography. After a detailed examination, PPV was performed for all patients who did not respond to other treatment options. All patients had a follow-up at 1, 3, six, and twelve months postoperatively, carefully examining postoperative results and postoperative complications. All patients have signed informed consent for both the surgical procedure and the study participation.

The inclusion criteria for this research were: adult patients of both genders, diagnosis of PDR, indication for PPV after all other treatment options were unhelpful, and signed consent forms to participate in the study. The exclusion criteria were: previous vitrectomy, absence of diabetes, other ophthalmological conditions including glaucoma, uveitis, central retinal vein occlusion (CRVO), and branch retinal vein occlusion (BRVO), mental disability, acute or chronic inflammatory disease, the presence of malignancy, and refusal to participate in the study.

All patients had preoperative laboratory examinations, including complete blood count (CBC), differential blood count (DBT), erythrocyte sedimentation rate (ESR), blood glucose levels, and HbA1c. Preoperative baseline characteristics included: blood pressure, anamnestic data regarding duration of insulin intake, antihypertensive medication, previous PRP, HbA1c values, and intravitreal VEGF values. 

According to the standard surgery protocol, PPV was performed in local retrobulbar or general endotracheal anaesthesia using as standard either 20G or 23G Millenium or Stellaris, respectively. The surgical field was prepared with 10% Iodine, setting the surgical drape, adjusting the blepharostat, and rinsing the conjunctiva with 3% Iodine. Sclerotomies were positioned above and below lateral and above medial rectus muscle on 3.5- or 4-mm posterior to the limbus, depending on the lens status. If the eye was phakic, phacoemulsification (PHACO) was performed with intraocular lens implantation and closure of main and port-incisions with 10-0 prolene suture. After the insertion of the irrigation cannula, light, and vitrectomy cutter through sclerotomies, vitreous and retinal inspection followed. All pathological structures were removed, including proliferation, blood, membranes with or without internal limiting membrane (ILM) peeling, and focal endolaser photocoagulation. The surgery was finished by internal tamponade with air, expanding gas (SF6 or C3F8), or silicon oil. Sclerotomies were sutured using 7-0 vicryl suture and subconjunctival Garamycin injection was applied. Conjunctiva was again rinsed with 3% Iodine, blepharostat removed, and the bandage set.

Intraoperative variables included: blunt dissection, sharp dissection, gas injection, and combined cataract + PPV surgery. They were all recorded after the surgery for each patient. Postoperative variables recorded one day after surgery included: the presence of vitreous haemorrhage, fibrin exudation, iris rubeosis, glaucoma, and retinal re-detachment.

The study was performed according to the tenets of the Declaration of Helsinki. The institutional review board approved this research (February 24th, 2014., decision number 0207-8095). 

Statistical analysis was performed using the SPSS 16.0 software (IBM, Chicago, USA). The Kolmogorov-Smirnov test tested the distribution of variables. The values were presented as the means ± standard deviations (SDs). One-way analysis of variance (ANOVA) was used to evaluate the changes in the BCVA. Univariate and multivariate linear regression analyses were used to determine the significance of the association between final BCVA and independent variables. P-value <0.05 was considered statistically significant.

## Results

Linear regression analyses were performed to identify the potential predictors affecting the visual outcome after surgery. At first, possible predictors were classified into three categories, as follows: baseline preoperative variables, including gender, age, glucose, HbA1c, systolic pressure, diastolic pressure, IOP, hypertension, previous PRP, and baseline BCVA (**Table 1**); intraoperative variables, including blunt dissection, sharp dissection, gas injection, and combined PHACO+PPV (**Table 2**); and postoperative complications, such as vitreous haemorrhage, fibrin exudation, iris rubeosis, and retinal re-detachment (**Table 2**).

**Table 1:** Baseline preoperative variables

Variables	
Gender (Male/Female)	31 (51.7%)/29 (48.3%)
Age (years)	60.48 ± 9.63
Blood glucose level (mmol/L)	9.15 ± 2.92
HbA1c (%)	8.51 ± 1.76
Duration of insulin intake (years)	14.76 ± 10.02
Systolic pressure (mmHg)	160.83 ± 22.64
Diastolic pressure (mmHg)	90.66 ± 11.22
Intraocular pressure (mmHg)	17.35 ± 5.79
Intravitreal VEGF concentration	501.94 ± 229.76
Hypertension (years)	44 (73.3%)
Previous PRP	30 (50.0%)
BCVA	0.03 ± 0.05
*VEGF = vascular endothelial growth factor, PRP = panretinal photocoagulation, BCVA = best corrected visual acuity	

**Table 2:** Intraoperative and postoperative variables (one-day post PPV)

Intraoperative variables		Postoperative variables	
Blunt dissection	39 (65.0%)	Vitreous haemorrhage	19 (31.7%)
Sharp dissection	27 (45.0%)	Fibrine exudation	22 (36.7%)
Gas injection	25 (41.7%)	Iris rubeosis	12 (20.0%)
Combined PHACO + PPV	27 (45.0%)	Glaucoma	10 (16.7%)
		Retinal re-detachment	1 (1.7%)
*PHACO = phacoemulsification, PPV = pars plana vitrectomy			

Preoperative BCVA was 0.03 ± 0.05. Six months postoperatively, it was increased to 0.18 ± 0.19 and was significantly higher compared to the preoperative value (p<0.001). Twelve months after PPV, it was 0.20 ± 0.19, which was again significantly higher compared to both preoperative (p<0.001), and 6-month postoperative value (p=0.029) (**Fig. 1**). 

**Graph 1:**
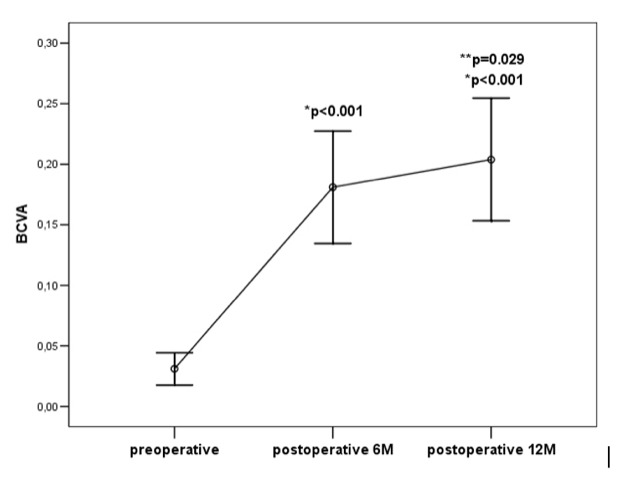
Changes of best-corrected visual acuity (BCVA) after vitrectomy *BCVA = best corrected visual acuity

After univariate linear regression analysis, glucose, HbA1c, VEGF, previous PRP, baseline BCVA, gas injection, vitreous haemorrhage, iris rubeosis, and glaucoma were found to be statistically significant parameters associated with postoperative visual outcome (p=0.004, <0.001, <0.001, <0.001, <0.001, 0.001, 0.015, 0.001 and 0.025, respectively) (**Table 3**). 

Multivariate linear regression analysis was performed to evaluate the association between these significant factors and postoperative visual acuity. Only intravitreal VEGF concentration, previous PRP, and gas injection remained statistically significant associated with postoperative BCVA (p=0.034, 0.012, and 0.020 respectively) (**Table 3**).

**Table 3:** Correlation of preoperative, intraoperative, postoperative variables and BCVA twelve months after PPV secondary to PDR, based on univariate and multivariate linear regression analysis

	Univariate linear regression analysis		Multivariate linear regression analysis	
	Coefficient	P-value	Coefficient	P-value
Preoperative factors				
Gender	0.002	0.990		
Age (years)	-0.165	0.208		
Blood glucose level (mmol/L)	-0.371	0.004	-.0143	0.150
HbA1c (%)	-0.637	<0.001	-0.161	0.250
Duration of insulin intake	0.009	0.947		
Systolic pressure (mmHg)	-0.039	0.765		
Diastolic pressure (mmHg)	-0.114	0.385		
Intraocular pressure (mmHg)	-0.172	0.189		
Intravitreal VEGF concentration	-0.734	<0.001	-0.335	0.034
Hypertension	-0.101	0.443		
Previous PRP	0.758	<0.001	0.413	0.012
BCVA	0.439	<0.001	0.172	0.056
Intraoperative variables				
Blunt dissection	-0.195	0.134		
Sharp dissection	-0.245	0.059		
Gas injection	0.445	<0.001	0.310	0.020
Combined PHACO+ PPV	0.032	0.477		
Postoperative variables (one-day post PPV)				
Vitreous haemorrhage	-0.314	0.015	-0.184	0.164
Fibrin exudation	-0.207	0.113		
Iris rubeosis	-0.403	0.001	-0.371	0.072
Glaucoma	-0.289	0.025	-0.049	0.799
Retinal re-detachment	-0.116	0.376		
*BCVA = best corrected visual acuity, PPV = pars plana vitrectomy, PDR = proliferative diabetic retinopathy, VEGF = vascular endothelial growth factor, PRP = panretinal photocoagulation, PHACO = phacoemulsification				

## Discussion

Advanced PDR with an indication for vitreoretinal surgery is always a challenging case to manage with an uncertain outcome. Most of these patients have very low BCVA preoperatively. Our study showed mean BCVA 0.03 ± 0.05, with a significant improvement at six months (0.18 ± 0.19) and especially twelve months postoperatively (0.20 ± 0.19). This was in correlation with other studies showing a significant increase in visual acuity. Ricca et al. reported that 6% of patients had BCVA ≥ 0.5 preoperatively with an increase to 30% five years postoperatively [**9**]. Gupta et al. reported 60.5% of patients with ≥3 ETDRS lines improvement and 38.38% of patients with increased final BCVA of ≥ 0.5 [**10**]. Higher BCVA compared to our study could be due to poor diabetic control of our patients, skipping regular follow-ups, and probable delay in surgeries due to a limited number of daily performed surgeries in the public sector with a single vitreoretinal surgeon. These factors could all contribute to the further progression of PDR changes.

Different factors can influence the final postsurgical visual outcome. Our study has divided these factors into preoperative, intraoperative, and postoperative. Preoperative variables included demographic and systemic factors. Variables related to poor visual outcome were older age, high blood glucose levels, HbA1c, higher systolic and diastolic blood pressure and intraocular pressure, hypertension, and higher intravitreal VEGF concentration. Improvement in visual outcome was observed in patients with a longer duration of insulin intake, previous PRP, and higher baseline BCVA. This indicated that the patients’ poor and inadequate systemic regulation of both diabetes and hypertension led to a higher incidence of complications and worse postoperative anatomical and visual outcomes. Most of these factors could be corrected by improving lifestyle, regular follow-ups, and adequate systemic therapy. Many other studies also reported systemic factors in relation to PDR. Gutpa et al. reported some predictors of poor visual outcomes such as longer duration of DM, insulin intake, delayed surgery, cardiovascular disease, and irregular follow-ups [**10**]. Schreuer et al. also reported higher age, lower baseline BCVA and TRD as an indication for lower postoperative visual acuity, but did not show the association of systemic factors with visual prognosis [**1**]. Previous completed PRP was a positive correlation with final BCVA in other studies as well, especially in young patients, due to a higher incidence of neovascular glaucoma because of ischemic PDR pathology [**11**]. Khan et al. reported PRP to be the most important PDR treatment in India [**12**]. They also reported that despite PRP, a third of the patients still needed PPV surgery, which was mostly due to incomplete sessions of PRP.

Intraoperative variables included blunt and sharp dissection, gas injection, and combined PHACO and PPV. Intraoperatively, both sharp and blunt dissection are correlated to worse visual outcomes. Patients who needed these surgical techniques have had fibrovascular membranes developed, which indicated more serious and advanced proliferative changes in the eye, which could be considered a negative predictor for visual outcome. Nishi et al. suggested that the absence of rubeosis and fibrovascular membranes resulted in BCVA ≥ 0.5, while the absence of rubeosis and the presence of vitreous haemorrhage preoperatively resulted in even higher BCVA ≥ 0.6 [**3**]. Used as internal tamponade, gas injection was proved to have a better visual outcome. Deiss et al. reported better functional outcomes with gas tamponade compared to silicon oil [**13**]. Most patients in this study had cataracts, which were due to diabetes and increased age. Vitrectomy also increased the incidence of cataract formation [**14**]. Therefore, almost half of the patients in our study underwent combined PHACO and PPV surgery, which resulted in better visual outcomes, making combined surgery a positive predictor for visual outcomes. Schreuer et al. also reported significant benefits after combined surgery [**1**]. 

Postoperative factors can influence visual outcomes during PPV. Our study showed a negative correlation between the presence of vitreous haemorrhage, fibrin exudation, iris rubeosis, glaucoma, and retinal re-detachment postoperatively with BCVA, which meant that their absence resulted in a better final visual outcome. Our results were confirmed in other studies as well [**3**,**9**]. Ricca et al. reported that eyes with vitreous haemorrhage have a higher incidence of BCVA gain compared to other complications due to PDR [**9**]. All these complications are the result of significant retinal ischemia, leading to neovascular glaucoma and, overall poor visual outcomes [**15**]. Postsurgical vitreous haemorrhage is a frequent complication in these patients, but usually clears more rapidly compared to preoperative bleeding [**16**]. 

Postsurgical complications in PDR patients include epithelial defects, fibrine exudation, glaucoma, iris rubeosis, vitreous haemorrhage, or retinal re-detachment [**6**]. Our study showed a higher percentage of these complications compared to other studies, some of which needed additional vitreoretinal surgery.

The final visual outcome affects the patients’ socioeconomic impact on the country, emphasizing their driving and working abilities and dependence on other people for everyday activities. Even though the final BCVA in our study did not reach the value eligible for driving, it is still considered a significant improvement for everyday activities and independence. The limitation of this study was that the fellow eye was not considered, which might have been better for fulfilling driving criteria. 

The advantage of this study was that a single vitreoretinal surgeon performed PPV, which decreased bias significantly. The prospective nature of the study also decreased bias and improved its significance. It was also the first study performed in Bosnia and Herzegovina to report visual outcomes after PPV surgery secondary to PDR. The major advantage was the presence of systemic condition parameters. The limitation of the study was a low sample size, but it was essential to consider that our country is in a deficit of vitreoretinal surgeons, and only a certain number of surgeries can be performed annually. In addition, it can serve as a pilot study for more extensive research over a longer period or even a multicentric study comparing the results among other centers performing vitreoretinal surgeries, further proving the usefulness of mentioned variables. Another limitation was a short follow-up period that could be prolonged to two and five years postoperatively to achieve more adequate results. Another improvement could be made by following patients who have received anti-VEGF treatment preoperatively. Overall, this valuable study indicated different variables affecting visual outcomes after PPV that should be considered when planning these surgeries. That is especially important for monocular, young patients, and the working population. 

## Conclusion

This study provided insight into factors influencing visual outcomes after PPV secondary to PDR. Visual outcome in most patients was improved after the surgery and remained improved after a twelve-month follow-up. Factors correlated with a better visual outcome were good systemic control, previous PRP, low intravitreal VEGF concentration, younger age, intraoperative internal gas tamponade, combined PHACO and PPV surgery, and the absence of postoperative complications. Patients with PDR should have regular follow-ups to indicate the surgery on time, thus improving the final anatomical and visual outcomes.

**Conflicts of Interest statement**

The authors declare that there are no conflicts of interest. 

**Informed Consent and Human and Animal Rights statement**

Informed consent has been obtained from the patients included in this study. 

**Authorization for the use of human subject**

Ethical approval: The case report related to human use complies with all the relevant national regulations, institutional policies, is in accordance with the tenets of the Helsinki Declaration, and has been approved by the review board of the Veneto Eye Bank Foundation, Venice, Italy. 

**Acknowledgments**

None. 

**Sources of Funding**

None.

**Disclosure(s)**

None.
